# Efficacy of single‐balloon overtube for endoscopic submucosal dissection in the proximal colon: A propensity score‐matched analysis

**DOI:** 10.1002/deo2.58

**Published:** 2021-09-28

**Authors:** Hidenori Tanaka, Shiro Oka, Akiyoshi Tsuboi, Yuki Kamigaichi, Hirosato Tamari, Akihiko Sumioka, Yasutsugu Shimohara, Tomoyuki Nishimura, Katsuaki Inagaki, Yuki Okamoto, Sumio Iio, Ken Yamashita, Kyoku Sumimoto, Shinji Tanaka

**Affiliations:** ^1^ Department of Endoscopy Hiroshima University Hospital Hiroshima Japan; ^2^ Department of Gastroenterology and Metabolism Hiroshima University Hospital Hiroshima Japan

**Keywords:** colorectal ESD, proximal colon, single‐balloon overtube, perforation, scope operability

## Abstract

**Objectives:**

A single‐balloon overtube (SBO) can improve poor scope operability during colonic endoscopic submucosal dissection (ESD). We aimed to evaluate the clinical usefulness of SBO for ESD in the proximal colon and the predictive factors for cases in which SBO is useful.

**Methods:**

A total of 88 tumors located in the proximal colon resected by balloon‐assisted ESD (BA‐ESD) using SBO and 461 tumors resected by conventional ESD (C‐ESD) between June 2015 and November 2020 were considered. Seventy‐eight tumors each in the BA‐ESD and C‐ESD groups were matched by propensity score matching. ESD outcomes were compared between the groups, and a decision tree analysis was performed to explore the predictive factors for cases in which SBO is useful.

**Results:**

There were no significant differences between the groups in the major outcomes such as en bloc resection rate (95% vs. 99%, *p* = 0.17), R0 resection rate (92% vs. 96%, *p* = 0.30), mean dissection speed (16 mm^2^/min vs. 16 mm^2^/min, *p* = 0.53), and intraoperative perforation rate (5% vs. 6%, *p* = 0.73). Even when considering cases with poor preoperative scope operability, there were no significant differences between the groups. Comparison of tumors ≥40 mm in diameter between the groups confirmed that the intraoperative perforation rate was significantly lower in the BA‐ESD group than in the C‐ESD group (0% vs. 24%, *p* = 0.0188).

**Conclusion:**

SBO is useful for ESD of tumors ≥40 mm in diameter in the proximal colon to prevent intraoperative perforation, which usually has a long procedure time.

## INTRODUCTION

Colorectal endoscopic submucosal dissection (ESD) has been standardized as a minimally invasive treatment method for colorectal tumors.^1^, ^2^, ^3^, ^4^, ^5^, ^6^, ^7^ However, there are cases that still require a high degree of skill and specialized techniques. Scope operability along with anatomical features such as wall thinness, folds, and flexures can affect technical difficulty.^8^, ^9^, ^10^ Poor scope operability, especially in the proximal colon, is caused by factors related to physique, intestinal adhesion, and respiration or heartbeat. Poor scope operability is a risk factor for incomplete resection and perforation.^8^, ^9^ The usefulness of various devices and methods that compensate for poor scope operability has been reported.^11^, ^12^, ^13^, ^14^, ^15^, ^16^, ^17^ Single‐balloon overtube (SBO) is one such assistive device. We previously reported that SBO can improve poor scope operability during ESD in the proximal colon,^18^ and there are other reports on its clinical usefulness.^19^, ^20^, ^21^ Kuroki *et al*. compared single‐balloon‐assisted ESD (BA‐ESD) and conventional ESD (C‐ESD) with poor scope operability and reported that SBO use improves the R0 resection rate for lesions from the cecum to the descending colon and dissection speed for lesions in the cecum or ascending colon.^21^ However, a problem of selection bias was latent, which possibly influenced the ESD outcomes, and it was uncertain in which cases SBO is useful and what its impact on treatment outcomes are.

In this study, we aimed to evaluate the clinical usefulness of SBO for ESD in the proximal colon and to explore the predictive factors for the cases in which SBO is useful.

## METHODS

### Patients

**FIGURE 1:**
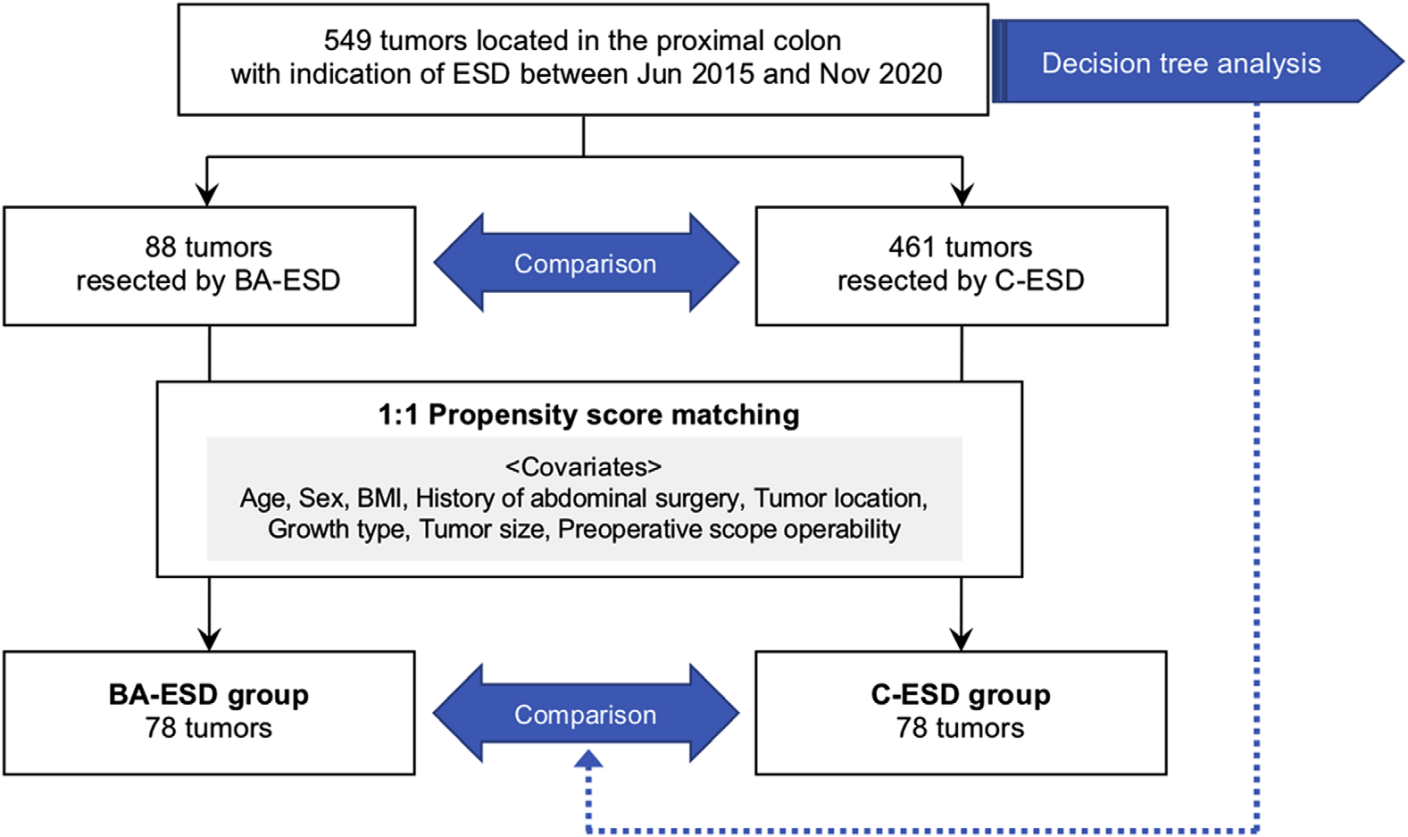
Flowchart of patient enrollment. ESD, endoscopic submucosal dissection; BA‐ESD, balloon‐assisted ESD; C‐ESD, conventional ESD; BMI, body mass index

Of 549 consecutive tumors located in the proximal colon, including on the ileocecal valve or appendiceal orifice, with an indication for ESD at Hiroshima University Hospital between June 2015 and November 2020, patient records of 88 tumors resected by BA‐ESD using SBO and 461 tumors resected by C‐ESD were reviewed. BA‐ESD was performed by four experts. Although SBO was preferred for poor preoperative scope operability cases, the decision to use SBO was at the endoscopist's discretion after referring to the preoperative data. To reduce selection bias, propensity score matching with a 1:1 ratio was conducted, with the caliper width set to 0.20, and 78 tumors in the BA‐ESD group and 78 tumors in the C‐ESD group were matched (Figure 1). The following items were set as covariates for propensity score matching: age, sex, body mass index (BMI), history of abdominal surgery, tumor location, growth type, tumor size, and preoperative scope operability.

This study was conducted in accordance with the Declaration of Helsinki and the study protocol was approved by Hiroshima University's Institutional Review Board (No. E2350).

### Indications for ESD

Indications for ESD, as described in the current guidelines published by the Japan Gastroenterological Endoscopy Society,^22^, ^23^ were as follows: lesions for which en bloc resection with snare endoscopic mucosal resection was difficult to perform, including laterally spreading tumor‐nongranular type, lesions showing type V_I_ pit pattern, carcinomas with shallow submucosal (T1) invasion, large depressed‐type tumors, and large protruded‐type lesions suspected to be carcinomas; mucosal tumors with submucosal fibrosis; sporadic tumors in conditions of chronic inflammation such as ulcerative colitis; and local residual or recurrent early carcinomas after endoscopic resection.

### ESD procedure

A colonoscope, either PCF‐Q260AZI (Olympus Medical Systems Co., Tokyo, Japan) or PCF‐H290TI (Olympus), was used. A standard tip hood (Olympus), ST hood (FUJIFILM, Tokyo, Japan), or its short type (FUJIFILM) was attached to the tip of the colonoscope. For submucosal injection, 0.4% sodium hyaluronate (Muco Up; Boston Scientific, Tokyo, Japan) diluted twice with a 10% glycerin solution was used. Of the three devices DualKnife (Olympus), DualKnife J (Olympus), or ITknife nano (Olympus), one or two devices were used as appropriate in each case. For BA‐ESD cases, SBO (ST‐CB1; Olympus) connected to a balloon control unit (OBCU; Olympus) was placed onto a colonoscope before insertion.^18^ SBO is a single‐use device and is less expensive than the ESD knife used in this study.

### Evaluation

The BA‐ESD and C‐ESD groups were compared based on the following outcomes: degree of submucosal fibrosis,^24^ histology, procedure time, dissection speed, intraoperative scope operability, use of snaring, en bloc resection, R0 resection (pathologically identified negative horizontal and vertical margins), and adverse events (including intraoperative perforation, delayed perforation, and postoperative bleeding). Dissection speed (mm^2^/min) was evaluated among the cases without using snare and calculated as long specimen diameter (mm) × short specimen diameter (mm) × 0.25 × 3.14/procedure time (min, from the initial submucosal injection to the completion of dissection). Scope operability was classified as poor, fair, or good. Poor scope operability was defined as occurrences of paradoxical endoscope movement, poor control with intestinal adhesions, and passive movement of the lesion or endoscope due to the patient's respiration or heartbeat.^8^ Good scope operability was defined as the performance of detailed and smooth maneuvers upon direct transmission of hand operation. Fair scope operability was defined as the absence of a completely smooth performance but without any hindrance to operation. Preoperative scope operability was evaluated on a day prior to ESD. Postoperative bleeding was defined as any apparent bleeding or hematochezia or >2 g/dl decrease in blood hemoglobin concentration compared with the preoperative level.^25^

To explore the predictive factors for cases in which SBO would be useful, a decision tree analysis for en bloc resection, dissection speed, and intraoperative perforation was performed using data from the 549 tumors considered for the study. The predictive factors were analyzed and extracted automatically from the following items collected as preoperative information: age, sex, BMI, history of abdominal surgery, tumor location, growth type, tumor size, and preoperative scope operability.

### Histologic diagnosis

All resected specimens were fixed in 10% formalin buffer, sliced into 2‐mm sections, and examined under a microscope. Histologic diagnosis was categorized as adenoma, intramucosal (Tis) carcinoma, T1a carcinoma with submucosal invasion depth of <1000 μm, and T1b carcinoma with submucosal invasion depth of ≥1000 μm, according to the criteria of the Japanese classification of colorectal, appendiceal, and anal carcinomas.^26^

### Statistical analysis

JMP version 15.0 (SAS Institute Inc., Cary, North Carolina, USA) was used for propensity score matching, decision tree analysis, and other statistical analyses. Continuous variables were analyzed using Student's *t*‐test or Mann–Whitney *U* test, and qualitative variables were analyzed using Pearson's chi‐square test or Fisher's exact test. A *p*‐value of <0.05 was considered statistically significant.

## RESULTS

**FIGURE 2:**
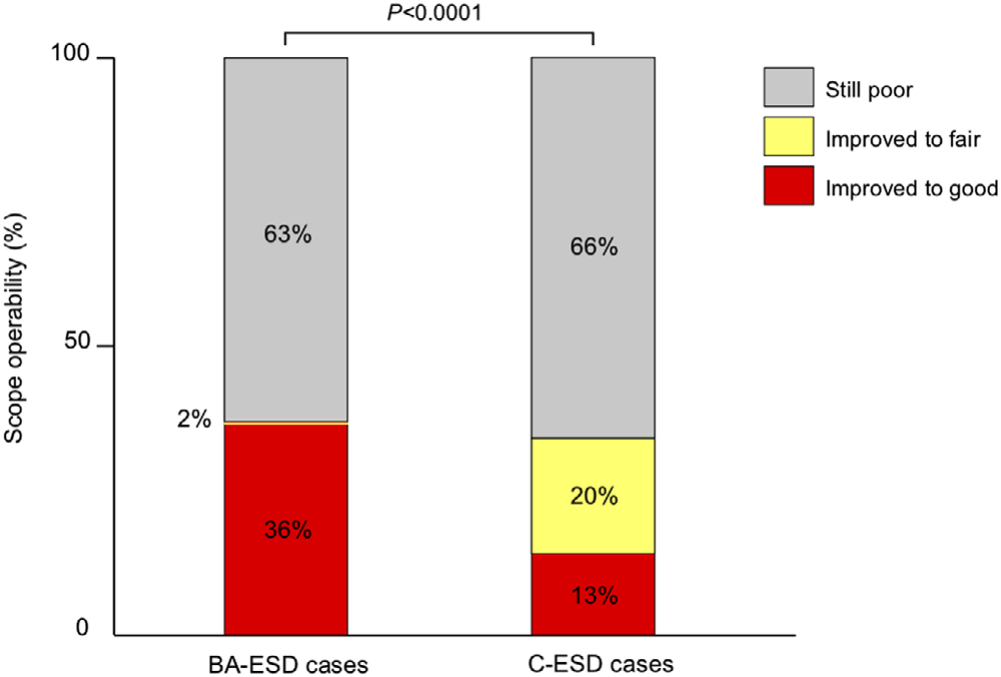
Changes in scope operability in cases with poor preoperative scope operability. Poor scope operability improved significantly in BA‐ESD cases compared with that in C‐ESD cases (36% vs. 13%, *p* < 0.0001). However, it was still poor in 63% of the cases despite SBO use. ESD, endoscopic submucosal dissection; BA‐ESD, balloon‐assisted ESD; C‐ESD, conventional ESD; SBO, single‐balloon overtube

Clinicopathological features and outcomes of 88 BA‐ESD and 461 C‐ESD cases are shown in Table 1. Four patients in C‐ESD cases underwent ESD for two lesions simultaneously. Patients in BA‐ESD cases were significantly older than those in C‐ESD cases (71 years vs. 68 years, *p* = 0.02). Mean tumor size was significantly larger in BA‐ESD cases than in C‐ESD cases (33 mm vs. 27 mm, *p* = 0.0003). There were significant differences between the groups in terms of tumor location and preoperative scope operability, that is, tumors located in the cecum/ascending colon with poor preoperative scope operability were more frequent in BA‐ESD cases than in C‐ESD cases. There were no significant differences between BA‐ESD and C‐ESD cases in terms of major outcomes such as en bloc resection rate, mean dissection speed, and adverse event occurrence rate; however, differences were observed in intraoperative scope operability and mean procedure time. Among the cases with poor preoperative scope operability, intraoperative scope operability significantly improved in BA‐ESD cases than that in C‐ESD cases (36% vs. 13%, *p* < 0.0001; Figure 2). Intraoperative scope operability was still poor in 63% of BA‐ESD cases despite SBO use.

**TABLE 1:** Clinicopathological features and outcomes of BA‐ESD and C‐ESD cases

**Variables**	**BA‐ESD (*n* = 88)**	**C‐ESD (*n* = 461)**	***p*‐value**
Age, year, mean ± SD	71 ± 11	68 ± 11	0.02
Sex, male (%)	54 (61)	287 (62)	0.87
BMI (kg/m^2^, mean ± SD)	24 ± 4	23 ± 4	0.06
History of abdominal surgery (%)	23 (26)	91 (20)	0.18
Tumor location (%)			0.0017
Cecum	28 (32)	111 (24)	
Ascending colon	45 (51)	181 (39)	
Transverse colon	15 (17)	169 (37)	
Tumor size (mm, mean ± SD)	33 ± 16	27 ± 12	0.0003
Growth type (%)			0.23
LST‐G	31 (35)	131 (28)	
LST‐NG	44 (50)	276 (60)	
Polypoid	13 (15)	54 (12)	
Preoperative scope Operability (%)			<0.0001
Good	5 (6)	85 (18)	
Fair	27 (31)	249 (54)	
Poor	56 (64)	127 (28)	
Colonoscope (%)			0.28
PCF‐H290TI	61 (69)	292 (63)	
PCF‐Q260AZI	27 (31)	169 (37)	
Submucosal fibrosis (%)			0.18
None/Mild	65 (74)	370 (80)	
Severe	23 (26)	91 (20)	
Histology (%)			0.11
Adenoma	41 (47)	274 (59)	
Tis carcinoma	35 (40)	129 (28)	
T1a carcinoma	4 (5)	25 (5)	
T1b carcinoma	8 (9)	33 (7)	
Procedure time (min, mean ± SD)	95 ± 72	68 ± 47	0.0033
Dissection speed (mm^2^/min, mean ± SD)	16 ± 10	16 ± 10	0.96
Intraoperative scope operability (%)			<0.0001
Good	31 (35)	117 (25)	
Fair	3 (3)	130 (28)	
Poor	54 (61)	214 (46)	
Use of snaring (%)	14 (16)	69 (15)	0.82
En bloc resection (%)	83 (94)	444 (96)	0.38
R0 resection (%)	81 (92)	433 (94)	0.51
Adverse event (%)			
Intraoperative perforation	4 (5)	26 (6)	0.68
Delayed perforation	1 (1)	1 (0)	0.19
Postoperative bleeding	3 (3)	10 (2)	0.48

Abbreviations: BA‐ESD, balloon‐assisted ESD; BMI, body mass index; C‐ESD, conventional ESD; ESD, endoscopic submucosal dissection; LST‐G, laterally spreading tumor granular type; LST‐NG, laterally spreading tumor non‐granular type; SD, standard deviation.

**FIGURE 3:**
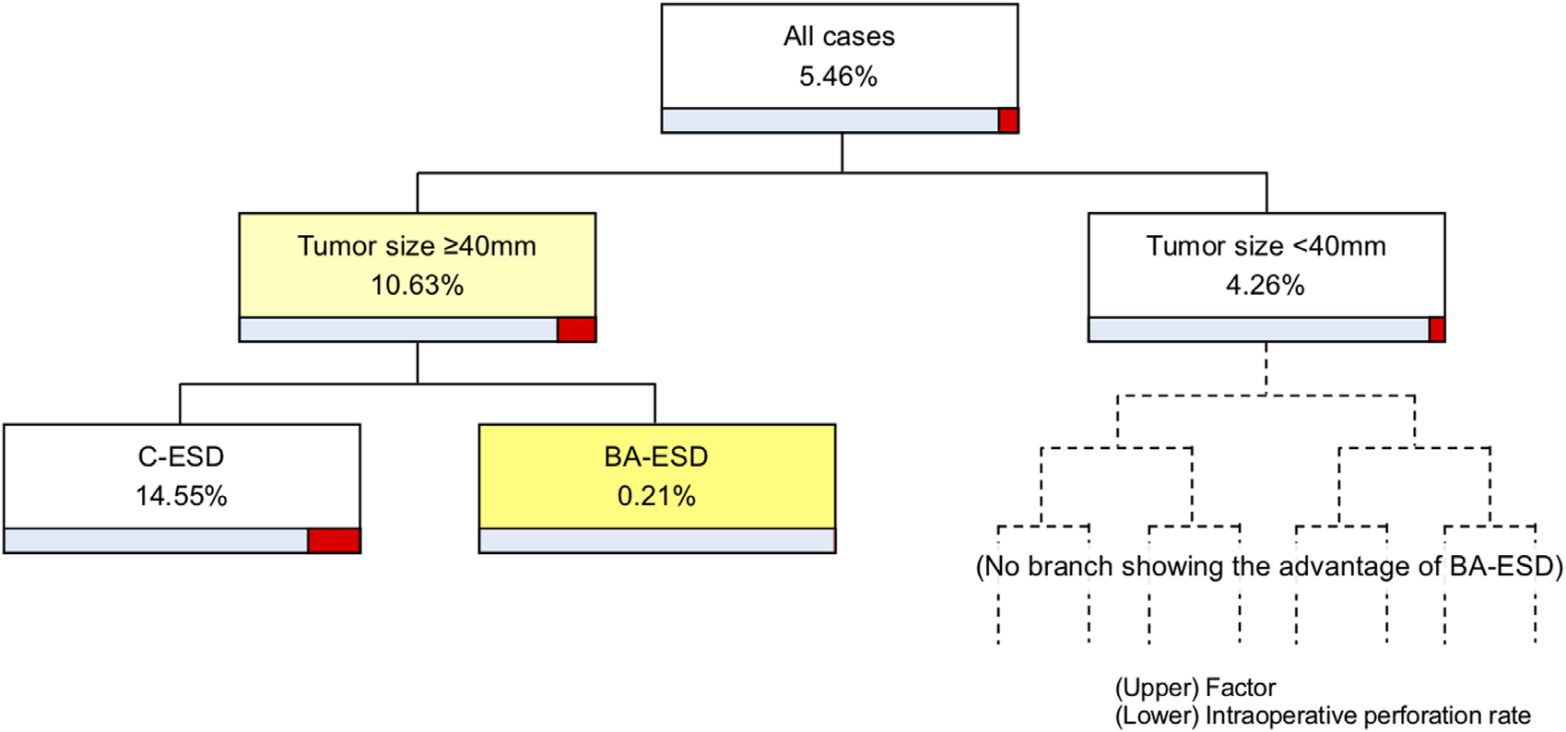
Decision tree analysis for preventing intraoperative perforation. Regarding tumors ≥40 mm in diameter, intraoperative perforation rates are 0.21% in BA‐ESD cases and 14.55% in C‐ESD cases. ESD, endoscopic submucosal dissection; BA‐ESD, balloon‐assisted ESD; C‐ESD, conventional ESD

Table 2 shows the clinicopathological features and outcomes of the BA‐ESD and C‐ESD groups after propensity score matching (caliper width = 0.2099). There were no significant differences between the groups in age, sex, BMI, history of abdominal surgery, tumor location, tumor size, growth type, and preoperative scope operability, all of which were covariates set in propensity score matching. There were no significant differences between the groups in terms of major outcomes such as en bloc resection rate (95% vs. 99%, *p* = 0.17), R0 resection rate (92% vs. 96%, *p* = 0.30), mean procedure time (87 min vs. 72 min, *p* = 0.24), mean dissection speed (16 mm^2^/min vs. 16 mm^2^/min, *p* = 0.53), and intraoperative perforation rate (5% vs. 6%, *p* = 0.73), except for intraoperative scope operability (*p* = 0.0006). Considering only cases with poor preoperative scope operability, there were no significant differences between the BA‐ESD and C‐ESD groups in en bloc resection rate (96% vs. 100%, *p* = 0.16), R0 resection rate (91% vs. 98%, *p* = 0.18), mean procedure time (91 min vs. 73 min, *p* = 0.37), mean dissection speed (16 mm^2^/min vs. 16 mm^2^/min, *p* = 0.74), and intraoperative perforation rate (9% vs. 9%, *p* = 0.97; Table 3). Endoscopist A performed 73% (64/88) of the BA‐ESD procedures (Table S1). For Endoscopist B, the rate of SBO use was low, mean dissection speed was slower, and en bloc resection rate was lower in the BA‐ESD group compared with the C‐ESD group. However, the difference disappeared after propensity score matching, and no significant difference was found in outcomes between the BA‐ESD and C‐ESD groups according to the experience of the endoscopist (Table S2). According to the decision tree analysis for en bloc resection rate and dissection speed, no predictive factors were observed that indicated the advantage of SBO use in BA‐ESD. On the other hand, the intraoperative perforation rate was low in BA‐ESD cases compared with that in C‐ESD cases for tumors ≥40 mm in diameter (0.21% vs. 14.55%; Figure 3). A comparison between the groups with tumors ≥40 mm in diameter confirmed that the intraoperative perforation rate was significantly lower in the BA‐ESD group than in the C‐ESD group (0% vs. 24%, *p* = 0.0188; Table 4).

**TABLE 2:** Clinicopathological features and outcomes of the BA‐ESD and C‐ESD groups after propensity score matching

**Variables**	**BA‐ESD (*n* = 78)**	**C‐ESD (*n* = 78)**	***p*‐value**
Age (year, mean ± SD)	70 ± 11	69 ± 11	0.78
Sex, male (%)	50 (64)	45 (58)	0.41
BMI (kg/m^2^, mean ± SD)	23 ± 4	23 ± 3	0.95
History of abdominal surgery (%)	19 (24)	23 (29)	0.47
Tumor location (%)			0.41
Cecum	27 (35)	29 (37)	
Ascending colon	36 (46)	40 (51)	
Transverse colon	15 (19)	9 (12)	
Tumor size (mm, mean ± SD)	31 ± 13	28 ± 13	0.23
Growth type (%)			0.72
LST‐G	25 (32)	28 (36)	
LST‐NG	42 (54)	37 (47)	
Polypoid	11 (14)	13 (17)	
Preoperative scope operability (%)			0.99
Good	5 (6)	5 (6)	
Fair	26 (33)	27 (35)	
Poor	47 (60)	46 (59)	
Colonoscope (%)			0.50
PCF‐H290TI	54 (69)	50 (64)	
PCF‐Q260AZI	24 (31)	28 (36)	
Submucosal fibrosis (%)			0.19
None/Mild	56 (72)	63 (81)	
Severe	22 (28)	15 (19)	
Histology (%)			0.88
Adenoma	39 (50)	40 (51)	
Tis carcinoma	28 (36)	24 (31)	
T1a carcinoma	4 (5)	5 (6)	
T1b carcinoma	7 (9)	9 (12)	
Procedure time (min, mean ± SD)	87 ± 64	72 ± 52	0.24
Dissection speed (mm^2^/min, mean ± SD)	16 ± 10	16 ± 9	0.53
Intraoperative scope operability (%)			0.0006
Good	25 (32)	17 (22)	
Fair	3 (4)	20 (26)	
Poor	50 (64)	41 (53)	
Use of snaring (%)	14 (18)	7 (9)	0.10
En bloc resection (%)	74 (95)	77 (99)	0.17
R0 resection (%)	72 (92)	75 (96)	0.30
Adverse event (%)			
Intraoperative perforation	4 (5)	5 (6)	0.73
Delayed perforation	1 (1)	0 (0)	0.32
Postoperative bleeding	3 (4)	1 (1)	0.31

Abbreviations: BA‐ESD, balloon‐assisted ESD; BMI, body mass index; C‐ESD, conventional ESD; ESD, endoscopic submucosal dissection; LST‐G, laterally spreading tumor granular type; LST‐NG, laterally spreading tumor non‐granular type; SD, standard deviation.

**TABLE 3:** Clinicopathological features and outcomes of poor preoperative scope operability cases

**Variables**	**BA‐ESD (*n* = 47)**	**C‐ESD (*n* = 46)**	***p*‐value**
Age (year, mean ± SD)	72 ± 9	71 ± 11	0.65
Sex, male (%)	30 (64)	28 (61)	0.77
BMI (kg/m^2^, mean ± SD)	23 ± 4	23 ± 3	0.51
History of abdominal surgery (%)	12 (26)	13 (28)	0.77
Tumor location (%)			0.43
Cecum	11 (23)	13 (28)	
Ascending colon	25 (53)	27 (59)	
Transverse colon	11 (23)	6 (13)	
Tumor size (mm, mean ± SD)	30 ± 10	30 ± 14	0.58
Growth type (%)			0.51
LST‐G	16 (34)	20 (43)	
LST‐NG	24 (51)	18 (39)	
Polypoid	7 (15)	8 (17)	
Colonoscope (%)			0.61
PCF‐H290TI	31 (66)	28 (61)	
PCF‐Q260AZI	16 (34)	18 (39)	
Submucosal fibrosis (%)			0.24
None/mild	34 (72)	38 (83)	
Severe	13 (28)	8 (17)	
Histology (%)			0.90
Adenoma	24 (51)	24 (52)	
Tis carcinoma	17 (36)	14 (30)	
T1a carcinoma	2 (4)	3 (7)	
T1b carcinoma	4 (9)	5 (11)	
Procedure time (min, mean ± SD)	91 ± 64	73 ± 44	0.37
Dissection speed (mm^2^/min, mean ± SD)	16 ± 11	16 ± 10	0.45
Intraoperative scope Operability (%)			0.02
Good	14 (30)	9 (20)	
Fair	1 (2)	10 (22)	
Poor	32 (68)	27 (59)	
Use of snaring (%)	9 (19)	5 (11)	0.26
En bloc resection (%)	45 (96)	46 (100)	0.16
R0 resection (%)	43 (91)	45 (98)	0.18
Adverse event (%)			
Intraoperative perforation	4 (9)	4 (9)	0.97
Delayed perforation	0 (0)	0 (0)	‐
Postoperative bleeding	1 (2)	1 (2)	0.99

Abbreviations: BA‐ESD, balloon‐assisted ESD; BMI, body mass index; C‐ESD, conventional ESD; ESD, endoscopic submucosal dissection; LST‐G, laterally spreading tumor granular type; LST‐NG, laterally spreading tumor non‐granular type; SD, standard deviation.

**TABLE 4:** Clinicopathological features and outcomes of the BA‐ESD and C‐ESD groups with tumor ≥40 mm in diameter

**Variables**	**BA‐ESD (*n* = 21)**	**C‐ESD (*n* = 17)**	***p*‐value**
Age (year, mean ± SD)	64 ± 15	70 ± 11	0.25
Sex, male (%)	16 (76)	11 (65)	0.44
BMI (kg/m^2^, mean ± SD)	24 ± 3	22 ± 2	0.06
History of abdominal surgery (%)	3 (14)	4 (24)	0.46
Tumor location (%)			0.30
Cecum	6 (29)	5 (29)	
Ascending colon	10 (48)	11 (65)	
Transverse colon	5 (24)	1 (6)	
Tumor size (mm, mean ± SD)	47 ± 14	47 ± 11	0.97
Growth type (%)			0.13
LST‐G	10 (48)	13 (76)	
LST‐NG	6 (29)	1 (6)	
Polypoid	5 (24)	3 (18)	
Preoperative scope operability (%)			0.21
Good	0 (0)	0 (0)	
Fair	9 (43)	4 (24)	
Poor	12 (57)	13 (76)	
Colonoscope (%)			0.13
PCF‐H290TI	15 (71)	8 (47)	
PCF‐Q260AZI	6 (29)	9 (53)	
Submucosal fibrosis (%)			0.95
None/mild	15 (71)	12 (71)	
Severe	6 (29)	5 (29)	
Histology (%)			0.95
Adenoma	8 (38)	7 (41)	
Tis carcinoma	8 (38)	5 (29)	
T1a carcinoma	1 (5)	1 (6)	
T1b carcinoma	4 (19)	4 (24)	
Procedure time (min, mean ± SD)	112 ± 73	119 ± 79	0.85
Dissection speed (mm^2^/min, mean ± SD)	20 ± 10	21 ± 12	0.72
Intraoperative scope operability (%)			0.53
Good	5 (24)	4 (24)	
Fair	0 (0)	1 (6)	
Poor	16 (76)	12 (71)	
Use of snaring (%)	2 (10)	0 (0)	0.19
En bloc resection (%)	19 (90)	16 (94)	0.68
R0 resection (%)	18 (86)	16 (94)	0.40
Adverse event (%)			
Intraoperative perforation	0 (0)	4 (24)	0.0188
Delayed perforation	0 (0)	0 (0)	‐
Postoperative bleeding	2 (10)	1 (6)	0.68

Abbreviations: BA‐ESD, balloon‐assisted ESD; BMI, body mass index; C‐ESD, conventional ESD; ESD, endoscopic submucosal dissection; LST‐G, laterally spreading tumor granular type; LST‐NG, laterally spreading tumor non‐granular type; SD, standard deviation.

## DISCUSSION

Propensity score‐matched analysis showed that major outcomes such as en bloc resection rate and adverse event occurrence rate in the BA‐ESD group were as good as those in the C‐ESD group, but the superiority of BA‐ESD was not observed. Even when only cases with poor preoperative scope operability were considered, the superiority of BA‐ESD was not observed, which is inconsistent with the finding of a previous report in which the R0 resection rate and dissection speed were favorable in BA‐ESD.^21^

A Difference in the duration between the groups within that study period may have led to inferior results in conventional ESD; however, we consider the reason to be that the R0 resection rate and dissection speed were favorable even in the C‐ESD group in this study, which were higher (98% vs. 83%) and faster (16 mm^2^/min vs. 11 mm^2^/min) than those reported in the previous report; further, the outcomes of the BA‐ESD group were as favorable as for those in the previous report.^21^ Decision tree analysis, which is a data mining method using machine learning, revealed that SBO is useful in preventing intraoperative perforation during ESD of tumors ≥40 mm in diameter located in the proximal colon. Although it is unknown how SBO prevents intraoperative perforation for large lesions, the degree of poor intraoperative scope operability could differ and SBO may slightly improve poor scope operability.

On evaluating outcomes by endoscopists, en bloc resection rate and dissection speed by Endoscopist B were worse in the BA‐ESD group than in the C‐ESD group although significant differences were not observed after propensity score matching. This was because Endoscopist B selectively performed ESD for cases with particularly poor scope operability, and poor preoperative scope operability was not improved with SBO in all cases.

SBO can improve scope operability by suppressing endoscope deflection, paradoxical movement, and passive movement of the lesion or the endoscope due to the patient's respiration or heartbeat. In addition, spontaneous deaeration through an overtube and the moderate collapse of the intestine improve scope operability and reduce patient distress. However, SBO cannot improve poor preoperative scope operability during ESD in all cases. Poor preoperative scope operability did not improve in 63% of cases even after using SBO; therefore, SBO should be recognized as a complementary, but not a revolutionary device. In addition, SBO is not required for all cases. ESD can be performed without SBO for small lesions or cases with good scope operability.

Yamashina *et al*. reported the usefulness of BA‐ESD using a double‐balloon endoscope (DBE) for tumors in the proximal colon with previous incomplete colonoscopies or unstable endoscopic maneuverability with conventional colonoscopes.^13^ The major outcomes of BA‐ESD using DBE were equal to those of conventional ESD, although there were no significant differences.

This study has some limitations. First, this was a retrospective study conducted at a single high‐volume center. Although propensity score matching was conducted to reduce selection bias, it could not be fully eliminated because SBO was used according to the endoscopist's discretion. In addition, the propensity score‐matched cohort in this study included more cases with poor scope operability than usual. A prospective trial is needed to resolve this issue. Second, scope operability was a subjective indicator assessed in the same manner as in previous reports,^8^, ^13^, ^18^, ^20^, ^21^ and preoperative and intraoperative conditions sometimes differ. Colonoscopes were also different between preoperative colonoscopy and ESD. In addition, endoscopists performing preoperative colonoscopy were different from those performing ESD. Establishing an objective indicator and further prospective studies are needed for a fair evaluation.

In conclusion, SBO helps improve the poor scope operability during ESD in the proximal colon in some cases but may worsen it in others. It is useful and can prevent intraoperative perforation for ESD of tumors ≥40 mm in diameter in the proximal colon, which usually has a long procedure time.

None

## CONFLICT OF INTEREST

The authors declare no conflict of interest for this article.

## FUNDING INFORMATION

None

## Supporting information




**Supplementary Table 1**. Outcomes of BA‐ESD and C‐ESD cases by endoscopists
**Supplementary Table 2**. Outcomes of BA‐ESD and C‐ESD groups after propensity score matching by endoscopists
