# Supplementary material for: Efficacy of single‐balloon overtube for endoscopic submucosal dissection in the proximal colon: A propensity score‐matched analysis
Source: DEN Open. 2021 Sep 28;2(1):e58. doi: 10.1002/deo2.58 (PMC8828212; doi:10.1002/deo2.58)
Supplement: Supplementary file 1 — Supplementary Table 1. Outcomes of BA‐ESD and C‐ESD cases by endoscopists Supplementary Table 2. Outcomes of BA‐ESD and C‐ESD groups after propensity score matching by endoscopists [file DEO2-2-e58-s001.docx]

Supplementary Table 1. Outcomes of BA-ESD and C-ESD cases by endoscopists

| Variables |  | Endoscopist A | | |  | Endoscopist B | | |  | Endoscopist C | | |  | Endoscopist D | | |
| --- | --- | --- | --- | --- | --- | --- | --- | --- | --- | --- | --- | --- | --- | --- | --- | --- |
|  |  | BA-ESD  n=64 | C-ESD  n=198 | *P*-value |  | BA-ESD  n=7 | C-ESD  n=144 | *P*-value |  | BA-ESD  n=12 | C-ESD  n=77 | *P*-value |  | BA-ESD  n=5 | C-ESD  n=30 | *P*-value |
| Tumor size, mm, mean±SD |  | 34±18 | 26±10 | 0.0003 |  | 37±8 | 30±13 | 0.044 |  | 27±10 | 26±16 | 0.31 |  | 25±14 | 22±6 | 0.81 |
| Preoperative scope operability (%) |  |  |  | <0.0001 |  |  |  | 0.0011 |  |  |  | 0.0012 |  |  |  | 0.80 |
| Good |  | 4 (6) | 31 (16) |  |  | 0 (0) | 35 (24) |  |  | 0 (0) | 14 (18) |  |  | 1 (20) | 3 (10) |  |
| Fair |  | 21 (33) | 112 (57) |  |  | 0 (0) | 63 (44) |  |  | 3 (25) | 45 (58) |  |  | 3 (60) | 21 (70) |  |
| Poor |  | 39 (61) | 55 (28) |  |  | 7 (100) | 46 (32) |  |  | 9 (75) | 18 (23) |  |  | 1 (20) | 6 (20) |  |
| Intraoperative scope operability (%) |  |  |  | 0.013 |  |  |  | 0.17 |  |  |  | 0.026 |  |  |  | 0.28 |
| Good |  | 26 (41) | 87 (44) |  |  | 0 (0) | 4 (3) |  |  | 3 (25) | 21 (27) |  |  | 2 (40) | 5 (17) |  |
| Fair |  | 1 (2) | 26 (13) |  |  | 0 (0) | 45 (31) |  |  | 1 (8) | 33 (43) |  |  | 1 (20) | 17 (57) |  |
| Poor |  | 37 (58) | 85 (43) |  |  | 7 (100) | 95 (66) |  |  | 8 (67) | 23 (30) |  |  | 2 (40) | 8 (27) |  |
| Dissection speed, mm^2^/min, mean±SD |  | 18±10 | 19±13 | 0.84 |  | 7±4 | 14±7 | 0.045 |  | 12±7 | 13±6 | 0.23 |  | 12±5 | 13±7 | 0.99 |
| En bloc resection (%) |  | 61 (95) | 192 (97) | 0.53 |  | 5 (71) | 135 (94) | 0.027 |  | 12 (100) | 76 (99) | 0.69 |  | 5 (100) | 29 (97) | 0.68 |
| R0 resection (%) |  | 59 (92) | 189 (95) | 0.31 |  | 5 (71) | 129 (90) | 0.14 |  | 12 (100) | 75 (97) | 0.57 |  | 5 (100) | 28 (93) | 0.55 |
| Adverse event (%) |  |  |  |  |  |  |  |  |  |  |  |  |  |  |  |  |
| Intraoperative perforation |  | 2 (3) | 10 (5) | 0.52 |  | 1 (14) | 11 (7) | 0.53 |  | 1 (8) | 4 (5) | 0.66 |  | 0 (0) | 1 (3) | 0.68 |
| Delayed perforation |  | 0 (0) | 0 (0) | - |  | 0 (0) | 0 (0) | - |  | 1 (8) | 1 (1) | 0.13 |  | 0 (0) | 0 (0) | - |
| Postoperative bleeding |  | 2 (3) | 7 (4) | 0.88 |  | 0 (0) | 3 (2) | 0.70 |  | 1 (8) | 0 (0) | 0.011 |  | 0 (0) | 0 (0) | - |

SD, standard deviation; ESD, endoscopic submucosal dissection; BA-ESD balloon assisted ESD; C-ESD, conventional ESD

Supplementary Table 2. Outcomes of BA-ESD and C-ESD groups after propensity score matching by endoscopists

| Variables |  | Endoscopist A | | |  | Endoscopist B | | |  | Endoscopist C | | |  | Endoscopist D | | |
| --- | --- | --- | --- | --- | --- | --- | --- | --- | --- | --- | --- | --- | --- | --- | --- | --- |
|  |  | BA-ESD  n=56 | C-ESD  n=27 | *P*-value |  | BA-ESD  n=5 | C-ESD  n=28 | *P*-value |  | BA-ESD  n=12 | C-ESD  n=17 | *P*-value |  | BA-ESD  n=5 | C-ESD  n=5 | *P*-value |
| Tumor size, mm, mean±SD |  | 31±14 | 29±12 | 0.49 |  | 36±9 | 31±16 | 0.21 |  | 27±10 | 24±7 | 0.24 |  | 25±14 | 24±6 | 0.48 |
| Preoperative scope operability (%) |  |  |  | 0.66 |  |  |  | 0.23 |  |  |  | 0.28 |  |  |  | 0.37 |
| Good |  | 4 (7) | 1 (4) |  |  | 0 (0) | 2 (7) |  |  | 0 (0) | 1 (6) |  |  | 1 (20) | 1 (20) |  |
| Fair |  | 20 (36) | 8 (30) |  |  | 0 (0) | 9 (32) |  |  | 3 (25) | 8 (47) |  |  | 3 (60) | 1 (20) |  |
| Poor |  | 32 (57) | 18 (67) |  |  | 5 (100) | 17 (61) |  |  | 9 (75) | 8 (47) |  |  | 1 (20) | 3 (60) |  |
| Intraoperative scope operability (%) |  |  |  | 0.0064 |  |  |  | 0.31 |  |  |  | 0.046 |  |  |  | 1.00 |
| Good |  | 20 (36) | 9 (33) |  |  | 0 (0) | 0 (0) |  |  | 3 (25) | 6 (35) |  |  | 2 (40) | 2 (40) |  |
| Fair |  | 1 (2) | 6 (22) |  |  | 0 (0) | 5 (18) |  |  | 1 (8) | 7 (41) |  |  | 1 (20) | 1 (20) |  |
| Poor |  | 35 (63) | 12 (44) |  |  | 5 (100) | 23 (82) |  |  | 8 (67) | 4 (24) |  |  | 2 (40) | 2 (40) |  |
| Dissection speed, mm^2^/min, mean±SD |  | 18±10 | 21±11 | 0.19 |  | 6±5 | 14±7 | 0.064 |  | 12±7 | 13±7 | 0.52 |  | 12±5 | 11±4 | 0.68 |
| En bloc resection (%) |  | 53 (95) | 27 (100) | 0.22 |  | 4 (80) | 27 (96) | 0.16 |  | 12 (100) | 17 (100) | - |  | 5 (100) | 5 (100) | - |
| R0 resection (%) |  | 51 (91) | 26 (96) | 0.39 |  | 4 (80) | 26 (93) | 0.36 |  | 12 (100) | 17 (100) | - |  | 5 (100) | 5 (100) | - |
| Adverse event (%) |  |  |  |  |  |  |  |  |  |  |  |  |  |  |  |  |
| Intraoperative perforation |  | 2 (4) | 0 (0) | 0.32 |  | 1 (20) | 4 (14) | 0.74 |  | 1 (8) | 1 (6) | 0.80 |  | 0 (0) | 0 (0) | - |
| Delayed perforation |  | 0 (0) | 0 (0) | - |  | 0 (0) | 0 (0) | - |  | 1 (8) | 0 (0) | 0.23 |  | 0 (0) | 0 (0) | - |
| Postoperative bleeding |  | 2 (4) | 1 (4) | 0.98 |  | 0 (0) | 0 (0) | - |  | 1 (8) | 0 (0) | 0.23 |  | 0 (0) | 0 (0) | - |

SD, standard deviation; ESD, endoscopic submucosal dissection; BA-ESD balloon assisted ESD; C-ESD, conventional ESD
